# A scoping review on weight loss injections and eating disorders: therapeutic impact, risks of misuse, and emerging harms

**DOI:** 10.1186/s40337-026-01634-6

**Published:** 2026-06-21

**Authors:** Poppy Barrett, Cat Papastavrou Brooks, Sara McCluskey, Amy Brown

**Affiliations:** 1 SPIRED Research Clinic, Sussex Partnership NHS Foundation Trust, Brighton, UK; 2 Sussex Eating Disorder Service, Sussex Partnership NHS Foundation Trust, Brighton, UK; 3 School of Psychology, University of Exeter, Exeter, UK

**Keywords:** Eating disorders, Binge eating disorder, GLP-1 receptor agonists, GIP agonists, Weight loss injections

## Abstract

**Aim:**

GLP-1 receptor and GIP agonists are widely used for weight loss in obesity, with strong evidence supporting their efficacy, and their potential positive impact on binge eating disorders. However, little is known about their use in individuals with eating disorders. Despite growing concerns, research exploring this intersection is limited, and clinical guidance is lacking.

**Objectives:**

This scoping review explores existing literature on weight loss injections in the context of eating disorder development and treatment to identify gaps and inform future practice.

**Methods:**

The scoping review searched six electronic databases (Embase, MEDLINE, PsycINFO, AMED, HMIC, and Emcare) in April 2025. Studies were eligible if they focused on the use of weight loss injections (GLP-1 receptor agonists), in individuals with current or past eating disorders, including all diagnoses. Both clinical and non-clinical settings were included, with no age or geographical restrictions.

**Results:**

A total of 80 records were identified through database searches. After title and abstract screening, 11 full-text articles were assessed for eligibility. Two papers were excluded due to the absence of empirical data, resulting in 9 studies being included in the final review.

**Conclusions:**

Overall, there is very limited evidence exploring the psychological impact of GLP-1 receptor agonists on individuals with eating disorders. While some findings suggest potential benefits for managing binge eating symptoms in populations with comorbid type 2 diabetes, there is a significant gap in our understanding of how these medications may influence disordered eating behaviours and body image concerns, particularly when used outside of weight-based indicators. Further research is essential to inform clinical guidelines.

**Supplementary Information:**

The online version contains supplementary material available at 10.1186/s40337-026-01634-6.

## Key points

GLP-1 RAs are increasingly used by people with eating disorders yet there is absence of clear clinical guidelines.

Evidence from randomized controlled trials, predominantly industry-sponsored, show potential benefit for binge eating disorder recovery.

The current evidence is methodologically weak and lacks qualitative research, indicating an urgent need for further study.

**Supplementary Information:**

The online version contains supplementary material available at 10.1186/s40337-026-01634-6.

## Abstract (highlights)

Weight loss injections are becoming increasingly popular, not only for diabetes and obesity but also among people with eating difficulties. Some early research suggests these medications might help reduce binge eating. However, very little is known about how these drugs affect people with other types of eating disorders, or how they may influence thoughts and behaviours around food, weight, and body image.

This review looked at all studies that examined weight-loss injections in people with eating disorders or disordered eating. Out of 80 studies found, only 9 met our criteria and most were a small sample or focused only on binge eating. Almost no research explored how these medications might cause or worsen eating disorder symptoms, and none looked at people using the injections without medical supervision.

Overall, the current evidence is very limited and cannot guide safe clinical practice. More research is urgently needed to understand both the potential benefits and risks for people with eating disorders or disordered eating.

**Supplementary Information:**

The online version contains supplementary material available at 10.1186/s40337-026-01634-6.

## Introduction

Weight loss injections or Glucagon-Like Peptide-1 Receptor Agonists (GLP-1 RAs) were originally created to treat type 2 diabetes [34]; they have since shown great potential in treating obesity [33]. They work by stimulating insulin secretion, inhibiting glucagon release, delaying gastric emptying and modulating neurological hunger, and satiety cues [35]. They have also been demonstrated to lower the risk of major cardiovascular events, such as heart attacks and strokes, in individuals with type 2 diabetes who have existing cardiovascular disease [17].

GLP-1 RAs have shown significant clinical benefits in the treatment of obesity and are approved for adults with obesity (BMI ≥ 30) or overweight (BMI ≥ 27), with at least one weight-related comorbidity [26]. They have quickly grown in public awareness and interest [29] due to their effectiveness on weight reduction in clinical trials, therefore creating a new prescription trend [3]. It is estimated that approximately 1.5 million people in the UK access weight‑loss injections privately [22], whereas fewer than 200,000 patients receive GLP‑1 injections for weight management through the NHS (NHS, 2025). A growing body of research highlights their efficacy in supporting weight loss and improving overall physical health [31]. As the evidence base for these medications expand, they are increasingly being integrated into clinical pathways for obesity and diabetes management.

However, far less is understood about the interaction between weight loss injections and eating disorders. According to the Diagnostic and Statistical Manual of Mental Disorder (DSM-5) eating disorders are serious illnesses marked by severe disturbances in a person’s eating with persistent disturbances of eating or eating-related behaviours leading to altered consumption, or absorption, of food which significantly impair physical health, or psychosocial functioning American Psychiatric Association, [15].

Eating disorders carry some of the highest mortality rates among psychiatric disorders, driven by both medical complications and elevated suicide risk [16, 65]. They are further associated with chronic psychological distress, reduced quality of life, and substantial impairment in daily and occupational functioning.

There are distinct clinical markers that differentiate eating disorder diagnoses (such as anorexia nervosa, bulimia nervosa and binge eating disorder), which guide clinical interventions and treatment pathways. However, many behaviours and core pathologies are shared across eating disorders, particularly concerning the overevaluation of weight and shape and maladaptive food-related behaviours [24]. People with eating disorders often oscillate between behaviours and it is not uncommon for one eating disorder to transition into another [27, 28, 32].

In clinical settings, there are growing concerns about use of the GLP-1 receptor agonists observed across all eating disorder diagnoses. Either prescribed by health care providers in accordance with national guidance or obtained via the private market, where often few safeguarding processes are in place. The risks associated with weight-loss injections span across all diagnostic groups, even those who may be deemed appropriate or clinically indicated for use. For example, individuals with BN or BED living with obesity may legitimately be prescribed WLIs with the desire for rapid weight loss, however, the fluid nature of these disorders may put them at risk of developing a more restrictive presentation [28, 30]. Those with more restrictive presentations, such as AN are at high risk (given the relative ease of obtaining the drugs) to misuse GLP1-RAs to maintain dangerously low body weight and further food refusal. Even in presentations not meeting full diagnostic criteria, the appetite-suppressing effects of WLIs may perpetuate disordered eating patterns.

Whilst GLP-1 receptor agonists and eating disorders have been observed to co-occur in clinical settings, there remains a lack of evidence-based guidelines to inform safe practice. This has raised growing concerns within ED services about the potential risks of individuals who are physically and mentally vulnerable accessing these medications, particularly via online advertisements and celebrity endorsements. Existing reviews on weight loss injections in eating disorders are limited, with systematic reviews focusing largely on studies investigating the use of GLP-1 receptor agonists in treatment for BED.

Several recent reviews have examined the potential role of GLP-1 receptor agonists (GLP-1RAs) in eating disorders. A systematic review and meta-analysis by Radkhah et al., [25] identified only five eligible studies, focusing largely on BED populations with higher BMI. While weight loss and reduced binge behaviours were reported as positive outcomes, the review lacked patient-centred perspectives, detailed sample characteristics, and broader reference to other ED presentations. Similarly, Aoun et al., [74] highlighted preliminary benefits but drew primarily from pilot studies, limiting generalisability and reinforcing the need for large-scale, placebo-controlled trials. Non-systematic reviews [19, 23] reported short-term improvements in binge eating and weight reduction, particularly with Semaglutide and Liraglutide, yet the evidence was based on a small number of trials with a narrow scope. Across these reviews, an overemphasis on weight-related outcomes and BED populations was evident, while long-term safety, effects in lower-BMI groups, and risks of heightening eating disorder psychological risks remain unclear.

### Aims

This scoping review aimed to map and summarize the existing literature on the use of weight-loss injections in the context of eating disorders. The following research questions were explored:

1. What is known about prescribed and unprescribed use of weight-loss injections among individuals with different eating disorder presentations and diagnoses?
2. What evidence exists regarding the relationship between weight-loss injections and the onset, maintenance, or exacerbation of eating disorder symptoms?
3. How do eating disorder services manage GLP-1 receptor agonist use, and what guidance, protocols, or tools are there?

## Methods

A scoping review was selected to map the emerging literature on this topic and identify gaps.

Our scoping review protocol was registered with the Open Science Framework (OSF). A SPFT subject librarian reviewed our research strategy and provided recommendations [13].

Methods and results are reported in line with PRISMA guidelines [14].

### Eligibility criteria

Studies were eligible for inclusion if they involved a population either with a diagnosed eating disorder or demonstrated symptoms of disordered eating and explored the use of weight loss injections including Semaglutide, Liraglutide, Tirzepatide, or other GLP-1 RAs. Both prescribed and non-clinically indicated use of these medications were included. All study designs were considered eligible. Only studies published in English were included.

Studies were excluded if they focused purely on weight loss medications administered in non-injectable forms, involved participants without any reference to eating disorders or disordered eating. Grey literature was excluded (See Table 1 for inclusion/exclusion criteria).

**Table 1:** Inclusion and exclusion criteria

Inclusion criteria	Exclusion criteria
Studies involving individuals with eating disorders, whether clinically diagnosed, assessed symptoms or self-reported	Grey literature: studies that are unpublished or not peer-reviewed (e.g., dissertations, conference abstracts, reports, etc.).
Studies specifically focusing on weight loss injections or pharmacological treatments intended for weight loss (e.g., semaglutide, liraglutide, GLP-1 receptor agonists, etc.)	Studies that focus on weight loss medications or treatments in non-injectable forms (GLP-1 patches, Orlistat)
Global research covering any geographical location or population	Studies involving individuals without a diagnosed eating disorder or without self-reported eating disorder symptoms (e.g., studies focused solely on obese populations without eating disorders).
Studies published in English.	Studies published in languages other than English
All study types, including randomized controlled trials (RCTs), cohort studies, case-control studies, observational studies, and qualitative studies.	Non-empirical Studies: theoretical papers, commentaries, editorials, opinion pieces, review articles, and position statements that do not present original empirical data
Both adult and paediatric populations (no age restriction).	
No restriction on study quality	
Clinically indicated/prescribed and non-clinically indicated use of weight loss injections	

### Search strategy

**Fig. 1:**
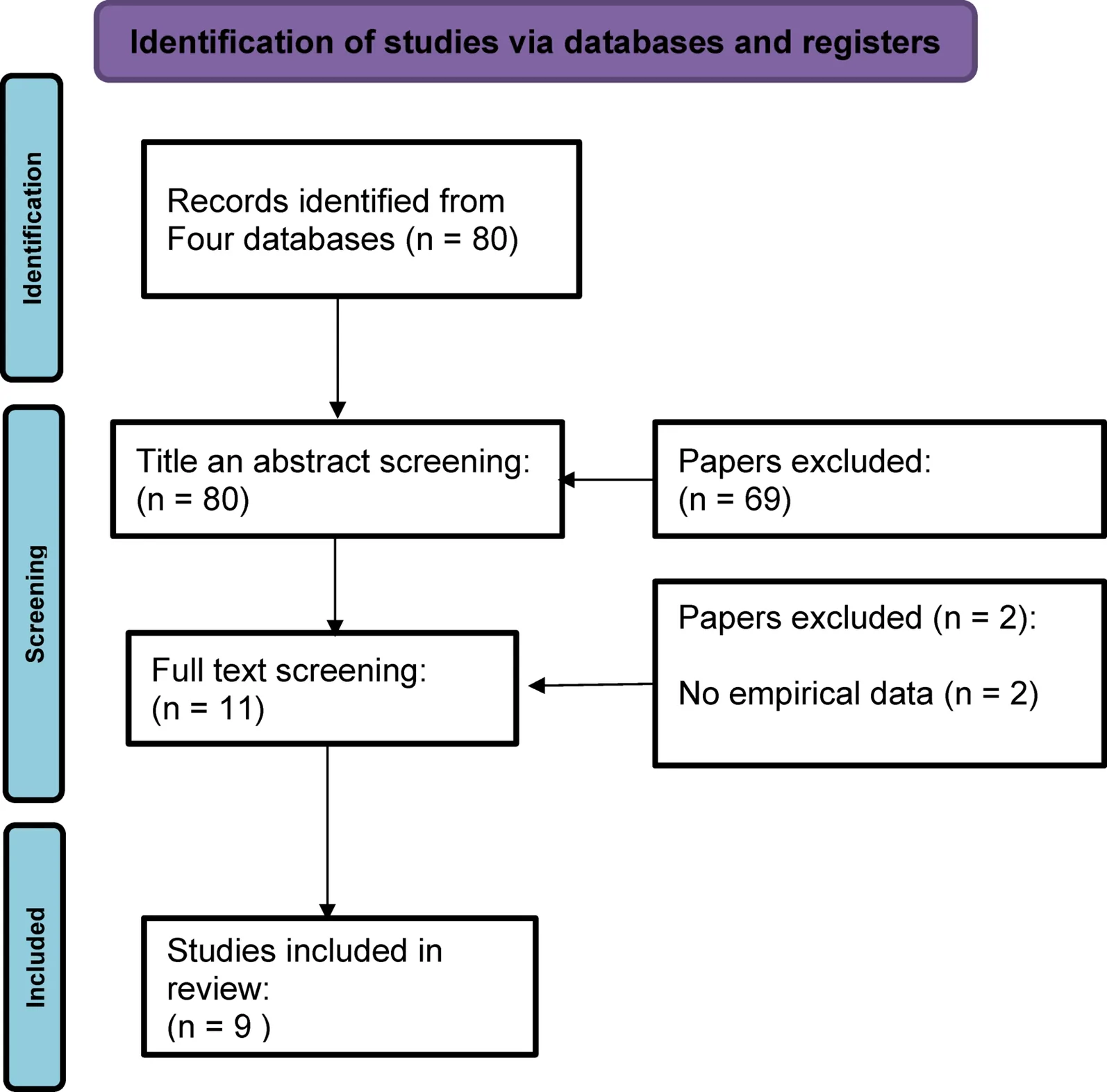
PRISMA 2020 flow diagram

A comprehensive search was conducted in April 2025 across six electronic databases: Embase, MEDLINE, PsycINFO, AMED, HMIC, and Emcare, using the OVID online database searching tool. The search strategy was developed to be as broad as possible to include all ED diagnosis and all weight loss injections. Terms related to eating disorders included “anorexia nervosa,” “bulimia nervosa,” “binge eating,” “ARFID,” “orthorexia,” “body image,” “disordered eating,” and related diagnostic classifications such as OSFED and UFED. Terms related to weight loss injections included “semaglutide,” “liraglutide,” “Ozempic,” “Wegovy,” “Saxenda,” “tirzepatide,” “Mounjaro,” “GLP-1,” and “weight loss injections.” Boolean operators (‘OR’ and ‘AND’) were used to combine search terms. (See Appendix 1 for full search strategy.) PRISMA flow diagram is included below (Fig. 1).

### Screening

Title and abstracts of all papers were retrieved and downloaded as a word document. All titles and abstracts were double screened by two reviewers (PB and CPB) acting independently, against the inclusion/exclusion criteria. All the full texts of included studies were also screened by two reviewers. Any conflicts were discussed and resolved through clarification of the inclusion/exclusion criteria.

### Extraction

Data was extracted from included studies by both researchers (PB) and (CPB) working independently, using a data extraction template including study information, sample demographics, main findings and weight loss injection (see Supplementary Material 1: Appendix 3 for the full extraction table.) Any discrepancies were resolved through meeting and discussion.

### Synthesis and quality assessment

No formal synthesis methodology was used; the aim of this scoping review was to summarize the studies used to evaluate any interactions between GLP-1 use and eating disorders and not assess effectiveness. No quality assessments were conducted.

## Results

A total of 80 records were identified through database searches. After title and abstract screening, 11 full-text articles were assessed for eligibility. Two papers were excluded due to the absence of empirical data, resulting in nine studies being included in the final review.

### Study characteristics

####  Study design

Across the nine included studies, several different study designs were used. Four studies reported on single or multiple patient cases to describe clinical presentations [7, 8, 11]. Two studies examined larger patient groups using retrospective reviews or cohort analyses [9, 12]. More rigorous designs included a randomized design by Allison et al., [1] and open-label pilot designs [5, 10]. Chen et al., [4] differed from the other designs and used data from the FDA Adverse Event Reporting System (FAERS) (Table 2).

**Table 2:** Study characteristics

Publication	Study design	Country	Conflict of interest	Funder	Setting
Seetharaman, S., & Cengiz, E. [11]	Case series	United States	No conflicts of interest	No funding	Single centre
Gong, J. Y., & Wentworth, J. M [7]	Single patient case report	Australia	Not reported	Not reported	Hospital (The Royal Melbourne Hospital)
Chen et al. [4]	Descriptive analysis of AE reports	China	No conflicts of interest	National Natural Science Foundation of China (Grant No. 82204758)	FDA Adverse Event Reporting System (FAERS) database
Guerdjikova et al. [8]	Case report	United States	No conflicts of interest	None	Specialist psychiatric treatment centre
Tempia Valenta et al. [12]	Retrospective clinical audit	Italy	Maria Letizia Petroni has received honoraria and participated in Novo Nordisk-sponsored studies	Open access funding by Università di Bologna	Hospital outpatient – Unit of Clinical Nutrition and Metabolism
Richards et al. [9]	Retrospective cohort study	United States	Dr. Richards is affiliated with Rhythm Pharmaceuticals and Novo Nordisk	None	Obesity medicine and bariatric surgery clinic
Allison et al. [1]	Pilot randomized controlled trial	United States	Multiple authors report associations with Eli Lilly, Novo Nordisk, WW International, Epitomee Medical	Novo Nordisk (Investigator-Initiated Study)	University of Pennsylvania
Da Porto et al. [5]	Pilot open-label, prospective controlled study	Italy	None	None	Outpatient diabetes service – Alto-Friuli-Collinare-Medio-Friuli
Robert et al. [10]	Pilot study, randomized prospective controlled trial	Malaysia	None	Universiti Kebangsaan Malaysia	Tertiary medical institution

#### Country

Four of the studies were conducted in the USA [8, 9, 11, 73], two were conducted in Italy [5, 12], one in Malaysia [10], and finally one in China [4].

#### Settings

The studies were conducted across a range of settings. Healthcare settings tending to be physical, not mental health, and included a hospital setting [7], a hospital outpatient unit [12], an outpatient diabetes service [75], an obesity medicine and bariatric surgery clinic [9], and one specialist psychiatric treatment centre [8]. One study Allison et al., [1] was conducted in a university setting, and one did not give information on the setting [11] just reporting that it was “single centre”. Chen et al., [4] analysed data from FAERS.

### Sample demographics: study sample

Many of the included papers had small sample sizes.

Tempia Valenta et al., [12] reported the largest intervention group with 54 participants, followed by Da Porto et al., [5] with 30 participants and Robert et al., [10] with 21 participants. Allison et al., [1] included 13 participants in the intervention group, while Seetharaman et al., [11] included 8 participants. Seetharaman and Cengiz [11] reported a sample size of eight patients, while two papers [7, 8] presented single patient case reports. Richards et al., [9] reported on data from 98 participants from an obesity medicine and bariatric surgery clinic [9]. Chen et al., [4] did not report a sample size as their analysis drew from the FAERS database.

Most studies included in this review did not have a control group. Of the three which did implement a control group, Allison et al. conducted a pilot RCT with 14 participants, Da Porto et al. used a prospective controlled design with 30 control participants, and Robert et al. included a control group of 21 participants receiving diet and exercise interventions. None of the three studies (Allison et al., Da Porto et al., Robert et al.) explicitly reported conducting a power calculation, raising concern about statistical power in the studies design (Table 3).

**Table 3:** Sample demographics

Publication	Sample Size	ED Diagnosis	Study Inclusion Criteria	Gender	Diabetes	BMI / Weight	Age	Race	Intervention group	Control group	Any other demographics	Clinical characteristics / co-morbidities	How they were sampled
Seetharaman, S., & Cengiz, E [11]	8	One patient developed an “eating disorder”	Adolescence and young adults (0-35) Type 1 diabetes (T1D) received GLP-RAs	F5 M3	Type 1	BMI – MEAN: 32.4, RANGE: 22.9–40.2, SD = 5.02	Age: MEAN = 19, Range: 26–13, SD = 5.37	Not reported	8	Not applicable	Not reported	Diabetes	Authors selected individual cases from their own clinical practice, not systematic
Gong, J. Y., & Wentworth, J. M [7]	1	Binge eating disorder	Type 1 diabetes (T1D)	F	Yes	BMI: 30.1 / 82 kg	27	Not reported	1	Not applicable	Not reported	Type 1 diabetes, obesity, severe binge eating disorder, anxiety and depression	Not reported
Chen et al. [4]	Not applicable	“eating disorder”	Not applicable	65.89% F, 30.96% M	Yes but unspecified	Unknown	All ages	Unspecified	No	No	No	Not reported	“FAERS is an openly accessible pharmacovigilance database documenting AEs, medication errors, and product quality complaints reported spontaneously by people of diverse occupations worldwide.”
Guerdjikova et al. [8]	1	Atypical Anorexia Nervosa	Not applicable	F	No	41.7	39	White	Not applicable	Not applicable	Voluntarily hospitalized for suicidal ideation on an inpatient general psychiatric unit in the fall of 2023	Pre-existing history of AN: diagnosed with severe, recurrent major depressive disorder, posttraumatic stress disorder, generalized anxiety disorder, and AAN	Identified through clinical practice
Tempia Valenta et al. [12]	54	Binge eating disorder	Adults (≥18) with BMI ≥30, or ≥27 plus ≥1 comorbidity, on liraglutide 3.0 mg by Aug 2022; excluded for incomplete assessment, non-adherence, or <2-month follow-up..	F40 M14	Type 2 diabetes	Mean BMI: was 39.89 (±6.05 SD) kg/m² ranging from 27.2 kg/m² to 59.5 kg/m²	The mean age was 48.56 (±13 SD) years	Unspecified	54	No	No	Approximately one-third of the patients had been prescribed psychotropic treatments while undergoing liraglutide therapy	Participants were not randomly assigned. Selected based on their existing treatment regimen
Richards et al. [9]	98	Binge eating disorder	Unknown	F80 out of 98 were women	No	No BMI just weight	Overall Mean: ≈ 43.8 years Overall SD: ≈ 12.5 years Overall Range: 21–79 (Range = 58 years)	White – 70, Black 6, American Indian 10, 2 Latino, 1 multiracial	No	No	No	All participants overweight / obese	Convenience sampling
Allison et al. [1]	27	Binge eating disorder	Adults with binge eating disorder (BED) diagnosed by DSM-5 criteria. Body mass index (BMI) of ≥ 27 kg/m² No diagnosis of type 1 or type 2 diabetes. Medically stable.	F17 M10	No	BMI: Average 37.9 SD: 11.8	Mean age 44.5 SD: 10.5	White 16, Black 11	13 Liraglutide	14	13 participants received liraglutide and 14 received placebo.	Twelve participants (44.4%) had a history of major depressive disorder, including three (11.1%) with a current episode; three (11.1%) had a current or past anxiety disorder.	Recruited via print, TV, radio, and online ads targeting those seeking BED treatment; screened by phone for interest and eligibility.
Da Porto et al. [5]	60	Binge eating disorder	Age 65, HbA1c between 7.5 and 9% on metformin therapy alone, normal renal function and diagnosis of BED	32F 28M	Yes, type 2 diabetes	Combined Mean = 34.4 Combined SD ≈ 6.17	Mean: 54.65 Combined SD = 7.76	Not reported	30	30	No	Type 2 diabetes, BED, obesity	Recruited in 2018 from the outpatient Diabetes service of AAS3 Alto-Friuli-Collinare-Medio-Friul.
Robert et al. [10]	44	showed binging behaviour	Presence of binge eating behaviour	Unknown	Not reported	Mean BMI 35.9 SD 4.2 kg/m²	Mean age: 34 SD: 9 years	Not reported	21	21: Diet and Exercise	Not reported	Non diabetic	Convenience sampling

#### Eating disorder diagnosis

Diagnosis varied across the studies. Five out of nine studies [1, 7, 9, 12, 75] only focused on participants with binge eating disorder (BED) diagnosis.

Seetharaman and Cengiz [11] described one patient who developed an unspecified “eating disorder.” Guerdjikova et al., [8] reported a single case of atypical Anorexia Nervosa. Finally, one study Robert et al., [10] institution described participants who engaged in binge-type behaviours but did not meet full DSM-5 criteria for BED. Chen et al., [4] categorized adverse event reports as “eating disorder” and did not specify.

#### Study inclusion criteria

Three studies specifically targeted populations with diabetes. Seetharaman and Cengiz [11] recruited young adults with type 1 diabetes (T1D) treated with GLP-1 receptor agonists, while Gong and Wentworth [7] reported a single T1D case. Da Porto et al., [75] focused on older adults with type 2 diabetes (T2D) on metformin therapy who also had co-occurring binge eating disorder (BED). In contrast, several studies examined eating disorder or obesity populations without a diabetes requirement, Chen et al., [4], Guerdjikova et al., [8], and Robert et al., [10] studied BED or binge eating behaviours in individuals without diabetes; Tempia Valenta et al., [12] and Richards et al., [9] explored obesity interventions where diabetes was not required, or not defined; and Allison et al., [1] included adults with BED but explicitly excluded those with T1D or T2D.

#### Gender

Two papers only had female patients ([7]; and Guerdjikova et al., [8]. Tempia Valenta et al., [12] participants were 74% female and 26% male (total sample 54). Richards et al., [9] reported that 82% of the sample were female and 18% were male (total sample 98). Allison et al., [1] recruited 27 participants of which 63% were female and 37% were male and Da Porto et al., [75] reported a more balanced sample with 53% female and 47% male (total sample 60). Seetharaman and Cengiz [11] reported 62.5% female and 37.5% male patients (total sample 8), and gender was not specified by Robert et al., [10]. Overall, most samples had a higher proportion of females. Finally, Chen et al., [4] reported adverse event cases with 65.9% female and 31.0% male representation.

#### BMI/Weight

Seetharaman and Cengiz [11] reported a mean BMI of 32.4 (range 22.9–40.2, SD = 5.02), while Gong and Wentworth [7] reported a BMI of 30.1 (82 kg). Guerdjikova et al., [8] included a patient with a BMI of 41.7, Tempia Valenta et al., [12] reported a mean BMI of 39.9 ± 6.05 kg/m², ranging from 27.2 to 59.5 kg/m². Allison et al., [1] included participants with a mean BMI of 37.9 ± 11.8, and Da Porto et al., [75] reported a combined mean BMI of 34.4 ± 6.17. Robert et al., [10] reported a mean BMI of 35.9 ± 4.2 kg/m². Richards et al., [9] reported weight only, without BMI. Chen et al., [4] did not provide BMI data. Overall, participants in these studies were predominantly in the overweight or obese range.

#### Age

Case series and reports featured younger individuals, with mean ages of 19 years (range 13–26, SD = 5.37; Seetharaman & Cengiz, [11]), 27 years [7], and 39 years [8]. The larger retrospective and prospective studies reported older mean ages: 48.6 years (SD = 13; [12]), (mean = 34 years, SD = 9; [10]), 43.8 years (SD ≈ 12.5; range 21–79; [9]), and 44.5 years (SD = 10.5; Allison et al., [1]).

Older populations were also represented, with mean participant ages of 54.7 years (SD = 7.8; [5]). No mean age values were provided in one study [4].

#### Race

The reporting of race and demographics was weak across the nine studies. Studies did not specify participants’ race or ethnicity [5, 7, 10, 11], whilst two studies described race in broad or unspecified terms [4, 12].

Among studies providing demographic data, Richards et al. reported a predominantly White sample (*n* = 70), with smaller representation of Black (*n* = 6), American Indian (*n* = 10), Latino (*n* = 2), and multiracial (*n* = 1) participants. Allison et al.‘s sample consisted of White (*n* = 16) and Black (*n* = 11) individuals. One case report identified the participant as White [8].

#### Any other demographics

No additional demographic information beyond age, race and gender was provided in the included studies.

#### Clinical characteristics and co morbidities

Seetharaman & Cengiz reported on adolescence and young adults with diabetes, while Gong & Wentworth described individuals with type 1 diabetes. Guerdjikova et al. included a patient with a history of anorexia nervosa (AN) and severe psychiatric comorbidities, including major depressive disorder, posttraumatic stress disorder, and generalized anxiety disorder. In the study by Tempia Valenta et al., approximately one-third of patients were prescribed psychotropic medications during liraglutide therapy. Richards et al. included only overweight or obese participants. Allison et al. reported that 44.4% of participants had a history of major depressive disorder, with 11.1% having a current depressive episode, and 11.1% having a historic or current anxiety disorder. Da Porto et al. included participants with type 2 diabetes, BED, and obesity, while Robert et al. studied non-diabetic individuals exhibiting binge eating behaviours. Chen et al. did not report any clinical characteristics.

BMI and weight data sat predominantly in the overweight to obese range. Seetharaman and Cengiz [11] reported a mean BMI of 32.4 (SD 5.02, range 22.9–40.2), while Gong and Wentworth [7] reported a BMI of 30.1/82 kg. Guerdjikova et al., [8] reported a BMI of 41.7. Tempia Valenta et al., [12] found a mean BMI of 39.89 (SD 6.05, range 27.2–59.5 kg/m²). Richards et al., [9] reported only weight data without BMI. Allison et al., [1] reported a mean BMI of 37.9 (SD 11.8). Porto et al., [5] reported a combined mean BMI of 34.4 (SD ≈ 6.17), and Rohana et al., (2015) reported a mean BMI of 35.9 (SD 4.2). Chen et al., [4] did not report BMI or weight.

#### Sampling method

Most studies relied on convenience sampling or existing patient populations. For example, Seetharaman and Cengiz [11] and Guerdjikova et al., [8] recruited participants from their own clinical practice, while Tempia Valenta et al., [12] selected individuals based on their current treatment regimen without random assignment. Richards et al., [9] and Robert et al., [10] also used convenience sampling, and Gong and Wentworth, [7] did not report their sampling method. In contrast, Allison et al., [1] deliberately recruited participants through advertisements in print, television, radio, and online, specifically targeting individuals seeking treatment for binge eating disorder (BED). Porto et al., [5] drew participants from an outpatient diabetes service.

Chen et al. sampled from FAERS between 2004 and 2023 to see how often people experienced mental health side effects while taking GLP-1 medications, researchers analysed patterns in these reports and compared how likely different psychiatric side effects were to occur with GLP-1 drugs compared to other treatments.

## Weight loss injections

### Medication used

Semaglutide was reported for use in four studies [7–9]; Seetharaman & Cengiz, [11] liraglutide three [1, 10, 12], Dulaglutide reported once [5] and one study which utilised a range of GLP-1 medications (Exenatide, Liraglutide, Lixisematide, Dulaglutide, Semaglutide and Tirzepatide) [4] (Table 4).

**Table 4:** Weight loss injection

Publication	Medication used	Dose	Provider	Duration	ED-Driven use of weight loss injections	Clinically indicated
Seetharaman, S., & Cengiz, E [11]	Liraglutide (Saxenda / Victoza), Semaglutide (Ozempic, Wegovy), or Tirzepatide (Mounjaro)	Variable doses	Hospital	5 months	No	Yes
Gong, J. Y., & Wentworth, J. M [7]	Semaglutide	Variable	Unspecified	10 months	No	Yes, obesity
Chen et al. [4]	Liraglutide, Semaglutide, Exenatide	Unspecified	Unspecified	Unspecified	No	
Guerdjikova et al. [8]	Semaglutide	Unspecified	2.4 mg	9 months (40 lb weight loss)	Yes	Unknown
Tempia Valenta et al. [12]	Liraglutide	3.0 mg	Novo Nordisk A/S	Unsure		Yes, then no
Richards et al. [9]	Semaglutide only (n=19); Semaglutide + another AOM (n=13); alternative AOM only (n=16)	Variable doses	Physicians at University of Oklahoma School of Community Medicine	Unspecified	No	Yes
Allison et al. [1]	Liraglutide	0.6 mg – 3.0 mg	Unknown	17 weeks		Yes
Da Porto, A et al. [5]	Dulaglutide	1.5 mg	Unknown	12 weeks	No	Yes
Robert et al. [10]	Liraglutide	0.623 mg – 1.8 mg	Unknown	12 weeks		Yes

### Dose

The studies reviewed reported a range of doses for weight loss injections. Two studies [7, 11] used variable doses. Tempia Valenta et al., administered 3.0 mg and Porto et al., used 0.6 mg to 3.0 mg, Allison et al., administered 1.5 mg, and Robert et al., used 0.623 mg to 1.8 mg. The three others [4, 8, 9] did not specify the dose.

### Provider

Seetharaman and Cengiz described the injection being provided within a hospital setting, while Richards et al. identified physicians at the University of Oklahoma School of Community Medicine as the provider. Tempia Valenta et al. stated that the intervention was supplied by Novo Nordisk, indicating industry involvement.

Studies by Gong and Wentworth, Chen et al., and Guerdjikova et al., reported unspecified provider details. In addition to this, studies by Allison et al., Da Porto et al., and Robert et al. did not report who provided the weight loss injection.

### Duration

Seetharaman & Cengiz administered treatment for 5 months, Gong & Wentworth for 10 months, and Guerdjikova et al. reported 9 months of therapy. Allison et al. received injections for 17 weeks, while Da Porto et al. and Robert et al. administered treatment for 12 weeks. The duration was unspecified in Chen et al. and Richards et al., and Tempia Valenta et al. did not clearly report the treatment length.

### Clinical indication

None of the studies examined the use of unprescribed, improperly prescribed, or illicitly obtained weight loss injections.

### Relationship between WLI and eating disorders

#### WLI being used as ED treatment

All included studies using WLIs as an eating disorder treatment focus on binge eating disorder or binge eating symptomatology, with no other eating disorder diagnosis explored. Gong and Wentworth, [7] reported remission, with a Binge Eating Scale (BES) score reduction from 39 to 3, alongside improved mood symptoms (DASS-21), maintained over 10 months. In contrast, Seetharaman and Cengiz [11] and Guerdjikova et al., [8] did not use GLP-1 RAs as ED treatment.

Valenta et al., [12] observed psychological and physical improvements in BED patients on GLP-1 RAs, However, patients with BED and co-occurring depression or anxiety were more likely to experience persistent binge eating even when continuing liraglutide for longer periods. Richards et al., [9] found Semaglutide produced significantly greater BES reductions than Lisdexamfetamine and topiramate, with sustained effects across moderate and severe BED groups (ANCOVA, F(2,92) = 10.1, *p*<.001).

Allison et al., [1] found no significant difference in binge eating remission between liraglutide (44%) and placebo (36%) at week 17 (*p* =.76), despite improvements in both groups. Porto et al., [5] and Robert et al., [10] reported significant reductions in BES scores following dulaglutide (*p* <.0001) and liraglutide (median 20→11, *p* <.001) (Table 5).

**Table 5:** Main findings

Publication	Main findings	Eating disorder as a consequence	WLI used as ED treatment	ED-Driven use of weight loss injections
Seetharaman, S., & Cengiz, E [11]	One case developed a restrictive eating disorder during study; Ozempic stopped after rapid weight loss of 44 lbs in 3.5 months. Restricted intake to 800 kcal/day and ran 30–60 min/day; aimed to lose 50 lbs. Referred to adolescent ED clinic.	Yes	No	No
Gong, J. Y., & Wentworth, J. M [7]	BES decreased from 39 to 3; DASS-21 improved at 2 months. At 10 months, binge eating remitted; depression, anxiety, and stress improved.	No	No	No
Chen et al. [4]	FAERS database: 181,238 AE reports for GLP-1 RAs; 8,240 (4.55%) psychiatric AEs. Eating disorder: 289 cases (ROR=1.57, 95% CI=1.40–1.77). Median onset 31 days (IQR 7–145.4). Exenatide longest (45 days), dulaglutide shortest (7 days), liraglutide (16 days), semaglutide (12 days), tirzepatide (10 days).	No	No	No
Guerdjikova et al. [8]	Weekly injections with energy drinks, supplements, laxatives, keto diet, intermittent fasting, excessive exercise. Rapid weight loss >16 kg (Feb–Oct 2022), >7 kg in weeks (Nov 2022). Severe GI pain; urgent cholecystectomy. Continued semaglutide until summer 2023; poor insight into AAN; persistent refusal to eat; weight/shape concern 9/10; desired weight 52 kg.	No	No	Yes
Tempia Valenta et al. [12]	Binge eating disorder showed psychological and physical improvements. Negative associations between anxiety (STAI) and MD, depression (BDI) and TD; higher likelihood of binge eating (BES>17) and TD. No significant correlation between BES and adherence indicators.	Potentially, clinically indicated	Yes	No
Richards et al. [9]	Semaglutide reduced BES scores significantly more than lisdexamfetamine and topiramate. Combined therapy did not improve BES beyond semaglutide alone. ANCOVA confirmed significant effect on BES: F(2,92)=10.1, p<.001.	No	No	No
Allison et al. [1]	Week 17: No significant differences in binge-eating remission between liraglutide and placebo (p=0.76). mITT: 44% liraglutide vs 36% placebo remission. Clinician ratings (CGII) “much improved” to “very much improved.”	No	No	No
Da Porto et al. [5]	Dulaglutide for 12 weeks: significant reduction in binge eating behavior (p<.0001; 12,067 vs 0,467).	No	No	No
Robert et al. [10]	Liraglutide participants: BES decreased significantly from 20 (IQR 18–27) to 11 (IQR 7–16), p<.001.	No	No	No

#### Eating disorder as a consequence of WLI use

Most included studies did not report the emergence of eating disorders as a consequence of GLP-1 receptor agonist (GLP-1 RA) treatment. However, two notable findings emerged. Firstly, Seetharaman & Cengiz [11] described a single adolescent case in which a restrictive eating disorder developed during Semaglutide (Ozempic) treatment. The patient lost 44 lbs within 3.5 months, reduced caloric intake to ~ 800 kcal/day, and engaged in daily exercise with a stated weight-loss goal of 50 lbs. The medication was discontinued, and the patient was referred for specialist eating disorder treatment.

Chen et al., [4] analysed 181,238 adverse event (AE) reports, 8240 (4.55%) were psychiatric AEs including 289 reports of eating disorders (reporting odds ratio [ROR] = 1.57, 95% CI 1.40–1.77). Median time to onset of psychiatric AEs was 31 days (IQR = 7–145.4 days), with nearly half (48.6%) occurring within the first 30 days. Median onset times varied by medication, from as short as 7 days with dulaglutide to as long as 45 days with exenatide.

The other studies consistently did not report evidence [1, 5, 7–9, 10, 12].

#### Eating disorder driven use of weight loss injections

Across the included studies, the majority did not report evidence of eating disorder–driven misuse of GLP-1 receptor agonists [1, 4, 7, 9, 5, 10, 11].

Guerdjikova et al. [8] reported one patient with a diagnosis of atypical anorexia nervosa (AAN) who was initially prescribed Semaglutide for weight loss, and experienced rapid weight loss as a result of the injections which exacerbated GI issues. They continued to abuse the medication alongside extensive compensatory behaviours which resulted in admission to a specialized mental health and eating disorder treatment centre.

In the retrospective audit by Tempia Valenta et al. [12], GLP-1 RAs were prescribed clinically for binge eating disorder (BED) management. While not explicitly driven by disordered eating intentions, the authors highlighted the potential for these medications to interact with existing psychopathology, given depression and anxiety were comorbid binge eating symptoms.

#### Conflicts of interest and funding

Conflict of interest and funding sources varied across the included studies, with several papers failing to clearly disclose support, raising concerns about transparency. Importantly, both randomised controlled trials (RCTs) included in this review were industry funded, primarily by Novo Nordisk, while the less robust case reports and observational studies reported no conflicts. This trend highlights the role of pharmaceutical companies in funding higher-quality trials compared to the relative independence of smaller scale studies.

Tempia Valenta et al., [12] reported no conflicts of interest, however, one of the co-authors disclosed receiving honoraria for lectures and advisory board participation from Novo Nordisk, in addition to participating in sponsored studies by the company. Richards et al., [9] reported that the lead author serves on the Speaker Bureau for Rhythm Pharmaceuticals and Novo Nordisk and sits on Rhythm Pharmaceuticals Advisory Board; the other authors reported no conflicts of interest. Additional industry ties were also noted among authors in other included studies [5].

## Discussion

This review aimed to explore the use of weight loss injections in the context of eating disorder development and treatment. We found some evidence that WLI could treat BED, with weight-loss effectiveness and some potential therapeutic benefit of GLP‑1 RAs for mood, cognition and binge eating behaviour [12] and reduction in binge eating frequency and improvement in weight outcomes [1]. However, none explored the lived experience of patients taking these medications. Critically, there was no research focusing on unprescribed/inappropriately prescribed WLIs, for example use in individuals with BMIs in the healthy or even underweight ranges. Consequently, there is a significant gap in understanding how services are responding to, monitoring, or supporting individuals with eating disorders who are utilizing weight loss injections.

The limited reporting of race and ethnicity across the studies included in our review highlights a significant gap in understanding how weight-loss injections may interact with eating disorder (ED) risk across all populations, limiting the generalizability of our findings. Ethnicity is an important consideration as ethnic groups experience higher medical risks related to obesity which could influence patient safety and experience outcomes of weight-loss interventions.

### Summary of findings

This review highlights an emerging body of evidence indicating the potential of WLIs as part of an effective treatment programme for BED. Allison et al., [1] found behavioural weight‑loss interventions combined with medication can reduce binge eating frequency and improve weight outcomes in patients with BED. Our findings support the literature that GLP-1 treatments appear to reduce the “food noise” experienced by individuals with BED, decreasing impulsivity around food while on the medication. In addition to this semaglutide appeared to help with BED symptoms and obesity and diabetes simultaneously [7] showing weight loss in this population can improve diabetes outcomes and reduce obesity-related health risks. This links to existing review findings (Radkhah et al.,2025, 74] and demonstrates the possible importance of pharmaceutical involvement when treating BED alongside a cognitive treatment which may be required for psychological support [8, 9]. There is a recognition that GLP‑1 RA initiation should be avoided in T1D patients due to an increased risk for restrictive eating behaviours [11], however the transitions between binge-eating and restrictive type ED presentations makes this recommendation complex to implement in practice [27, 28].

Some included papers looked at the development of EDs because of WLI use, though these were predominantly small case reports, as well as a single study analysing data from the FDA Adverse Event Reporting System [4]. No studies in our review examined in depth the interaction between EDs and WLIs, particularly in the context of ED treatment. Given the limited evidence base for weight loss injections utilized as part of an ED presentation, parallels could be drawn with anabolic steroid use and their well-documented link with eating disorders and body dysmorphia [76]. Mounting evidence led to the classification of anabolic steroids as a class C drug, in part due to the abuse and misuse of these injections among vulnerable populations with body image disturbances [51, 76, 77]. Consequently, use of these medications may demonstrate short-term measurable effects, desirable to the user, while masking long-term physical and psychological harm [50, 54, 55]. While GLP-1RAs reduce food cravings or addictive eating behaviours (Amorim Moreira [36]; Amorim Moreira [36]), for someone with an eating disorder, the drive for weight loss and the tendency to engage in food avoidance behaviours may become the addictive element [42]. In other words, the medication’s mechanisms that support addiction in those without eating disorders may function as the addictive substance in this population [46]. Further research is required to explore risks due to the high potential for psychological dependence when utilising self-administered injectables.

### Strengths, limitations and directions for future research

#### Review method

This systematic review was limited by its design as a scoping review, meaning we did not undertake quality appraisals. However, no existing quality appraisal tools for systematic reviews explicitly include funder or conflict of interest considerations; a key factor compromising the objectivity of the included studies. The scoping design also meant we did not utilize a formal synthesis method; however the limited number and heterogeneity of included studies would have precluded conducting a meta-analysis, and there were no qualitative studies included. Until there is an increase of primary research on weight-loss injections and eating disorders, it is unlikely to be worthwhile to conduct a full systematic review in this area.

Another methodological limitation of this review was that we did not examine grey literature, which may have led us to miss emerging evidence as the research base is rapidly evolving. In addition, exploring clinical service-level approaches to managing eating disorder presentations in the context of GLP-1 use could offer valuable insights into real-world practice. This is possibly the most significant limitation, as it is likely that services have developed internal analyses, protocols, or treatment frameworks that are not yet published in peer-reviewed journals. However, this was designed as a rapid scoping review, conducted by clinicians within a service setting, with the aim of keeping pace with emerging evidence to inform clinical practice.

We included only English-language papers, which may have led to some omissions; however, as most high-quality research in this area is conducted by pharmaceutical companies, such omissions are likely to be few.

The main strength of this review is that the search strategy and inclusion criteria were structured to identify not just the use of GLP-1 injections to treat binge-eating disorder, as with other systematic reviews [25, 74] and non-systematic reviews [23], but to understand how they might interact with eating disorders in a clinical context, including inappropriate use of GLP-1 injections. A factor influencing the aims, methods, and overall approach of this review was the composition of the research team. The review was led by an Senior Assistant Psychologist (PB) working in a UK community eating disorder service, who frequently treats patients using or misusing GLP-1 injections. The co-authors included Consultant Psychologist (AB) and Clinical Director for Adult Eating Disorders (SM) within the same service, as well as a mixed-methods researcher with a background in public health and mental health research (CPB). Regular reflexive discussions were held between PB, CPB, and AB regarding how their positionalities shaped the review process. These discussions acknowledged that the review was undertaken from the standpoint of an immediate clinical need for guidance in this area, alongside an emphasis on funding sources more commonly observed in public health research.

Future research should also address the prevalence of GLP-1 receptor agonist misuse within eating disorder populations, including the role of private prescriptions and unregulated access which currently lacks sufficient evidence despite emerging clinical concerns.

#### Included studies

The main limitation of this scoping review is the quantity and quality of included studies. In contrast to previous reviews [19, 23, 25, 74], we deliberately adopted a broad inclusion strategy (all locations, all EDs all WLI) and still identified very limited evidence, highlighting the substantial gaps in this area.

Many of the more methodologically robust studies included in our review were funded by large pharmaceutical companies, raising concerns about the introduction of potential bias ([59]; Wade et al.,2020 [72], [60]). There is an established relationship between pharmaceutical industry funding and biased research, including publication bias (non-publication of negative findings and multiple- publication of positive findings) poor methodological design (including choice of comparator and enriched enrollment) and the conclusions of trial publications not reflecting results [59]. Systematic reviews have highlighted a range of mechanisms through which the pharmaceutical industry exerts influence on the research they fund, including through ghost writing or ghost management of research, and the creation of social ties and obligations between industry funders and research scientists [68]. Parallels could be drawn between pharmaceutical industry funded research utilizing GLP-1 injections as a treatment for BED, and industry funded research into opioids in the 1990 s, where similar issues around distortion of findings, ghost-writing, and methodologically flawed studies, resulted in inaccurate claims around the safety, efficacy and non-addictiveness of opioids in treating pain [44, 71].

All included studies predominantly focused on weight-based outcomes, with none incorporating qualitative methodologies. Most studies relied on BMI as the main outcome measure, which has been criticized as an outdated and non-holistic metric, further undermining results [39, 45]. Evidence on intentional weight loss and all-cause mortality is mixed. Meta-analyses in general populations suggest no clear mortality benefit [47], though some RCTs show short-term reductions in mortality, particularly with obesity [57, 73]. Effects may also differ by metabolic status, with GLP-1 receptor agonists reducing cardiovascular risk in non-diabetic samples but showing different results in those with diabetes [78].

The papers frequently promote problematic messages around eating disorders, emphasizing weight loss as the primary marker of success. This narrow framing often overlooks psychological functioning, day-to-day impact, and broader health outcomes, subtly reinforcing the notion that thinness is synonymous with positive outcomes, regardless of the methods used to achieve it [2, 58, 67]. This approach limits understanding of the complex psychological aspects of eating disorders, which extend beyond measurable weight data (Peláez-Fernández et al., 2022) [64]. There was a lack of consideration in included papers of how GLP-1 injections might interact with underlying psychological factors such as trauma, body dysmorphia, distress surrounding loose skin/skin elasticity changes, depleted muscle mass, emotional regulation and other psychosocial complexities [62], or in clinically valid research, including qualitative work to give detailed patient perspectives or studies exploring long-term harms [66].

The short-term nature of many of these studies is a factor as well, reduction in one off binge-eating symptoms reported does not necessarily equate to long term, meaningful recovery [56] and the timelines of these studies are nonrepresentative of real-world recovery interventions [48, 49]. Generalisability is also limited, as study populations were small, with restricted diversity in terms of race, setting, and socio-demographics.

This pattern may be partly explained by the influence of funding sources (particularly pharmaceutical and obesity-focused interests) which is reflected in the fact that nearly all included studies were conducted in physical health settings, with one exception, which took place in a specialist psychiatric clinic. Many of these studies were funded by the pharmaceutical industry and sought to expand the application of GLP-1 treatments beyond obesity and diabetes into psychological or behavioural domains. This raises concerns about the reliability and generalisability of their outcomes for informing real-world eating disorder treatment, were harm minimisation and meaningful, patient-centred outcomes should be central [52, 63]. Consequently, there is a pressing need for more exploratory, qualitative, and complex research conducted by those with psychiatric expertise, ideally through publicly funded initiatives that prioritise mental health perspectives and a more comprehensive understanding of patient experience and recovery [40].

All studies looked at clinically prescribed use of GLP-1 injections. Although research on illicit use is limited, there is evidence of negative health consequences from people gaining access to them through others’ prescriptions, online pharmacies Frisbie, [43], or “off label” use by bodybuilders [70]. However, no included papers address this phenomenon.

### Clinical implications

GLP-1 receptor agonists show preliminary promise for binge eating disorder (BED), with reports of reduced binge episodes and improved metabolic outcomes. However, evidence remains confined to small studies and case reports, offering limited insight into broader health or psychological effects. Patients with BED often present with emotional vulnerabilities and trauma histories, where binge eating functions as a coping mechanism. Consequently, once “healthy” weight goals are achieved, use of these medications may shift toward misuse if underlying psychological needs remain unmet.

NHS services are encountering patients across all diagnoses and weights who are accessing or misusing these medications. Many patients face financial barriers, as GLP‑1 injections are costly, which can influence adherence, continuity of care, and the risk of unsupervised use.

Given the psychological complexity of eating disorders, screening prior to and during GLP-1 use may be warranted. The Eating Disorder Examination Questionnaire (EDE-Q) and the weekly Eating Disorder-15 (ED-15) help clinicians capture eating disorder cognitions surrounding weight and shape concerns and behaviours. Outcomes such as these could support initial risk assessment and support ongoing monitoring and determine psychological suitability for treatment.

Future research should examine the full spectrum of eating disorder symptoms and associated perceptual or cognitive disturbances that may emerge among GLP-1 users. This should include restrictive eating patterns, self-induced vomiting, compulsive or compensatory exercise, body and weight checking behaviours, excessive food tracking, and body image disturbances, including body dysmorphic concerns.

Overall, current evidence offers little clinical guidance. Reported symptom improvements lack long-term physical or psychological follow-up, and none of the studies explored interventions for weight loss injection misuse. Eating disorders are also reduced to weight or symptom-based outcomes, often overlooking their psychological, social, and emotional complexity. This makes it difficult to draw meaningful lessons for real-world clinical practice.

### Conclusion

The current literature on GLP-1 receptor agonists in eating disorder populations remains limited and methodologically variable, with some of the research informed by industry funding. Evidence shows that interventions targeting weight loss in binge-eating disorders can lead to meaningful physical health improvements, particularly for diabetes and other obesity-linked outcomes. In addition to this binge eating symptoms have shown to decrease whilst on GLP-RAs. However less is attention given to the psychological, social, and emotional factors that contribute to a range of eating disorder presentations. Research in heterogeneous, complex, high-risk populations would benefit from greater emphasis on combined multimodal approaches, for example, integrating pharmacological interventions such as GLP-1 receptor agonists or fluoxetine with evidence-based psychological therapies. Many studies rely on small case reports or industry sponsorship, which provide limited clinical guidance for NHS services seeking evidence-based approaches which are person centred at heart. Key areas for further exploration include qualitative and long-term follow-up data, ethical considerations, and validated measures of psychological impact. The use of binge eating scales introduces some variability due to the ongoing debate over identifying objective versus subjective binge eating behaviours, reflecting the broader complexities in clinical assessment and intervention.

Despite the increasing public use of these medications and widespread promotion online and via social media, evidence to support safe, patient-centred clinical practice is still emerging. Much of the current research is US-based and industry-funded, which may limit generalisability, and data from specialist centres, or regulatory reporting systems offer only partial perspectives. While some interventions show reductions in discrete behaviours, the broader implications for health, functioning, and recovery remain underexplored. Eating disorders are complex mental health conditions that require robust, detailed, assessment and specialist psychological interventions. Pharmacological treatments may play a supportive role, but alongside evidence-based psychological interventions as to not overlook the psychological mechanisms necessary for full recovery.

Overall, the literature highlights an important opportunity for rigorous, independent research that addresses both psychological, physical and social outcomes, supporting clinicians and services in providing safe, effective care for individuals with complex eating disorders.

## Supplementary Information

Supplementary Material 1.

## Data Availability

No datasets were generated or analysed during the current study.

## Appendix 1

### Key terms search list

#### Category 1: eating disorders

- Eating disorder
- Eating disorders
- Disordered eating
- Eating-related difficulties
- Feeding and eating disorder
- Dysfunctional eating behaviours
- Maladaptive eating patterns
- Body image
- Anorexia Nervosa
- Bulimia Nervosa
- Binge eating
- Avoidant Food Intake Disorder
- Restrictive Food Intake Disorder
- ARFID
- Body dysmorphia
- Body perception
- Rumination Disorder
- Other Specified Feeding or Eating Disorder
- OSFED
- Unspecified Feeding or Eating Disorder
- UFED
- Orthorexia
- Underweight
- Low weight

#### Category 2 : weight loss injections

- Weight loss injections
- Semaglutide
- Ozempic
- Wegovy
- Liraglutide
- Saxenda
- Tirzepatide
- Mounjaro
- Setmelanotide
- IMCIVREE
- GLP-1
