# Supplementary material for: A scoping review on weight loss injections and eating disorders: therapeutic impact, risks of misuse, and emerging harms
Source: J Eat Disord. 2026 Jun 21;14:194. doi: 10.1186/s40337-026-01634-6 (PMC13491749; doi:10.1186/s40337-026-01634-6)
Supplement: Supplementary file 1 [file 40337_2026_1634_MOESM1_ESM.docx]

Data Extraction Template

This template was developed to systematically extract study characteristics, sample demographics, intervention details, and eating disorder–related outcomes for the purposes of this scoping review.

**Study Information**

| Study Number |  |
| --- | --- |
| Publication Date |  |
| Authors |  |
| Study Design |  |
| Country |  |
| Conflict of Interest |  |
| Funder |  |
| Setting (Community / Hospital / Other) |  |
| Sampling Method |  |

**Sample Characteristics**

| Sample Size |  |
| --- | --- |
| Eating Disorder Diagnosis |  |
| Inclusion Criteria (e.g., Diabetes?) |  |
| Gender Distribution |  |
| Diabetes Status (Type 1 / Type 2 / None) |  |
| BMI / Weight |  |
| Age (Mean, SD, Range) |  |
| Race / Ethnicity |  |
| Other Demographics |  |
| Clinical Characteristics / Comorbidities |  |

**Intervention Details**

| Intervention Group Description |  |
| --- | --- |
| Control Group Description |  |
| Medication Used (GLP-1 RA type) |  |
| Dose |  |
| Provider |  |
| Duration |  |
| Clinically Indicated (Yes / No / Unclear) |  |

**Outcomes and Findings**

| Eating Disorder as Consequence (Yes / No + Details) |  |
| --- | --- |
| Weight Loss Injection Used as ED Treatment (Yes / No + Details) |  |
| ED-Driven Use of Weight Loss Injection (Yes / No + Details) |  |
| Main Findings |  |
| Adverse Events Reported |  |
